# Book Reviews

**Published:** 1855

**Authors:** 


### REVIEWS.

### (A.—HISTORICAL AND CRITICAL.)

### SANITARY REGULATIONS IN THE BRITISH ARMY.*

If a Frenchman, German, or American, on visiting England four years ago, should have ventured to call in question the capabilities of the whole of our military system, and boldly to prophesy that, in the event of any great war in which the English nation might be engaged, the grossest mismanagement would of necessity result; if he had said, "You English people have in you no principles of action worthy of a magnificent warlike enterprise; you can neither victual an army nor a transport, you cannot conduct a siege operation, nor can you even keep up anything approaching to sanitary regulations amongst a body of men encamped some three thousand miles away on an open coast;" if, we say, a foreign visitor should have dared to call in question the abilities of the English nation on these points, there would, we believe, have been but one answer —*Only try us.* The Englishman, looking around him with conscious pride, would have thought of his country's naval resources, of its stalwart soldiers, of its admirable system of constitutional government, of its undying national courage, of its incalculable wealth, and of its past successes in arms. He would have thought of all these matters; and the doubt presented by the sceptical questioner of the majesty and power of England would have been received with simple contempt.

The events of the past ten months, however, have taught us the painful lesson, that an overweening spirit of pride and self-confidence, exercised now for some years past, has done not a little to sap the foundations of our power as a nation; and they moreover indicate, in broad outline, the fact that we can retain our position in the van of the world's march only by desperate efforts, which, in the absence of a complete reform, must soon fail, leaving us dishonoured and almost lost.

It would lead us away from the object of this essay, and would only give rise to the repetition of a tale a thousand times told, to

---

* Parliamentary Committee. Inquiry into the State of the Army before Sebastopol: 1855.

Hennen (John, M.D.). Principles of Military Surgery: 1820.

M'Grigor (Sir James). Medical Sketches of the Expedition to Egypt: 1804.

Pringle (Sir John). Observations on the Diseases of the Army: 1768.

De Kerckhoff (J. R. L.). Histoire des Maladies Observès à la Grande Armée: Anvers, 1836.

Bird (James, M.D.) Laws of Epidemic and Endemic Diseases, and Importance of Preventive Medicine: 1854.

Récueil de Mémoires de Médecine, de Chirurgie, et de Pharmacie Militaires, rédigé sous la surveillance du Conseil de Santé. 2me série, treizième volume. Paris: 1854.

enter into any description of the causes that have led to the system of disorder and anarchy that has lately prevailed in our army service. It concerns us only to look at the subject in relation to the health of the troops whom England has sent out, and to the provision that has been made for their social and their sanitary welfare.

In entering on this task, we desire to express merely that which we believe to be true, without any of that violent and rash declamation in which some more impulsive writers have freely indulged. We look upon the awful catastrophe in the East with as much horror as any one; but as it has occurred, and, so far as the past is concerned, is quite irremediable, let us, before the recollection of it becomes a mere gigantic shadow in history, apply to some practical purpose the lessons which it is fitted to teach, and try to distil out observingly the soul of good which is said to be ever present in things evil.

When the news first arrived in this country of the sufferings to which the English soldier was exposed, from the mere neglect of the first, the simplest sanitary regulations, the accounts received seemed to imply so direct a departure from common sense and common humanity, that the reading public were actually inclined to question the accuracy of the records placed in their hands. Nay, so powerfully has this feeling prevailed, that nothing less than the parliamentary inquiry, now being conducted, could have sufficed to satisfy the whole of the community of the general truth of all the appalling statements communicated from time to time by the correspondents of the newspaper press. The whole truth has, however, now come to the light, and admits—and, indeed, invites—free and thoughtful discussion.

The questions which are now on most lips, are:—Was the calamity, of which we have heard the history, inevitable? Was it the result of negligence? Was it the offspring of ignorance? Or, lastly, was it merely the indication of a vicious organisation, which, having its roots in old and base forms, set intellect at defiance, and paralysed the energies of competent and deserving men?

The evidence on these points seems to point out clearly a double cause—incompetency in individual men, inefficiency in the system by which military affairs are managed. To lay all blame on the system alone implies, at all events, a much more lenient bearing toward the acts of several of the more important persons concerned in the war, than the evidence given before Mr. Roebuck's committee will in any degree warrant. Let us then turn our attention to the disaster in its general bearings, and deal with this exclusively so far as it relates to sanitary regulations.

It is taking a very shallow view of the sanitary question in its relations to the army and navy services, to suppose that the disasters which our troops have recently suffered are strictly due, either to the effects of climate under unfortunate circumstances, or to the inefficiency of the organisation of the army medical system for the exigencies of active service. The simple truth is, that the sanitary measures followed out in the British forces are bad, both in peace and in war; and that no attempt of a systematic kind has ever been made to bring into active operation those hygienic laws, which science has long been endeavouring to define and to teach.

Nay, we may even take a wider view of the question now before us, and may trace the inefficiency of the sanitary regulations of our army to a similar inefficiency in every department of the empire, civil as well as military—an inefficiency which stares us in the face at every point, and in every waking hour of our lives. The fact is evident, demonstrative, that we, as a nation, are almost in the stages of barbarism as regards a correct application of the knowledge we possess of the laws relating to the preservation of health and life.

So strong, indeed, have been the prejudices of the British people against any kind of sanitary improvement, that not the simplest advance has been made in any one direction without violent and often, for some time, successful opposition. If a graveyard nuisance exist, and hundreds of lives yearly suffer from its influence, such nuisance can rarely, if ever, be removed or abated, without a loud and prolonged outcry from the very persons who feel the noxious element most severely. A variety of obstacles in such cases are almost invariably raised to prevent the innovation. One man has the right to a piece of ground in the graveyard, and is determined to assist, when dead, in poisoning his friends, rather than give up that to which he believes he has a fair claim. Another man urges the question of respect for the remains of his ancestors; as if such remains were really still lying like Nineveh stones, in the identical spot where they were originally placed, instead of having long ago been resolved, wholly or partially, into their primitive elements, and in that form diffused over the face of the earth. A third raises his voice against the whole plan of reform, not because he sees special objections to it, but because it is a trespass on the opinions which he has from early youth imbibed.

Again, if a young physician or surgeon, entering into life as a practitioner, should venture to put into practice any physiological or sanitary principles, which good general knowledge and mere common sense may have suggested to him, he becomes at once, not the supported, but the branded man. He is theoretical; he has experience in all matters but in the treatment of disease; and his neighbour next door, who likes his pleasant society, and holds him to be one of the most clever men with whom he has ever conversed, would yet be very sorry to trust his life in such a man's hands. Thus the poor fellow starves so long as he remains true to truth; and when at last, necessity getting the upper hand of honesty, he gives up with aching heart his scientific loves and his natural mental gifts, and when, assuming the air of a practical man, with a confident knowledge of every disorder, a dozen remedies for any special symptom, a vast deal of hard and incomprehensible language, and excellent tact, he adopts the whole system of that blue bottle routine with which the greatest science in the world is so often disgraced, he then, and not till then, finds a patient in his library or a fee in his coffer.

We mention this subject, not as a digression, but as having a direct bearing on the matter now in hand. The facts we have stated shew, not that the army specially is behind the day in sanitary knowledge, but that it represents only one section of a community in a position similarly anomalous. To the spirit of universal negligence of sanitary topics, sacrifices must from time to time be inevitably offered; and on our army has now fallen the unhappy lot to furnish its hecatombs.

Throughout the course of the war, comparisons have been made between the health of the French and of the English troops, and the cause of this difference has been supposed to consist in a difference of military organisation. This may be the fact to a certain extent, but it is far from being the whole fact. The grand difference is based on the circumstance that the French, as a nation, are some fifty or a hundred years a-head of us in their practical knowledge and application of the means of preserving health. In England, we have no standard literature of hygiene,—no work on this subject shewing the footprints either of genius or of industry. In France, first-class works of this kind are as common as biographies, histories, or elementary treatises on natural science. The French have their Dictionary of Hygiene; their records of sanitary improvements; and, in a quarterly work, entitled *Annales d'Hygiène Publique*, the choicest epitome that can be imagined on all subjects relating to health and life. Their system of medical education, moreover, provides for instruction in the principles of sanitary science. Many of their most leading medical practitioners are distinguished as authors on hygiene, and still more on general science; and, as it is felt amongst the rising generation of their Esculapian brotherhood, that the surest way to become an eminent practitioner is to become an eminent scholar, there is little or no temptation offered for any man to hold back, with all his might, the assistance which he may be capable of furnishing to universal knowledge.

The science of the preservation of health, being thus freely promulgated among all classes of the French nation, becomes naturally an integral part of the organisation of their army medical service. The principles of discipline, so necessary for an army, shew themselves as fully in the hospital as on the marching ground; and the medical officer is, in his way, kept up to as strict a practical mark, as is the drill serjeant. The French military medical officer, who holds a position in the service vastly superior to that held by his brother in the British army, must possess, likewise, a thorough acquaintance with a variety of subjects. He must know the best sites for hospitals; the readiest means of building temporary hospitals; the simplest and easiest plans for conveying wounded men; the number of men that can be tolerated in one ward; the clothing best suited to the constitution of the soldier at home and abroad; the diets which may be most easily prepared, and be most suitable under various conditions; and the amount of physical toil which is compatible with the steady enjoyment of health. With all this information, he combines a correct and most extended knowledge of the nature and treatment of actual disease. Lastly, in order to add greater efficiency to the French army medical system, the records of the medical service are regularly published under government authority; and every important suggestion or improvement is thus duly chronicled and preserved.

When all these facts are taken into account, it becomes at once obvious that no actual comparison can be made between the medical services of the French and the English armies. As regards the treatment of actual disease, an analogy may exist; and we doubt not that, as mere practitioners, the English medical officers are at least equal to the French. But in an expedition far away, the old proverb, that prevention is better than cure, assumes a meaning doubly precious; and a medical corps is of less than half its real use, if it is either incompetent, or is not left free to act in the establishment and in the carrying out of those rules upon which alone prevention depends.

We trace then the misfortunes that have befallen our army to a general, as well as to a special source; and so far as this is the case, the chiefs of the army have some kind of general excuse. It may be urged, first, that they could not foresee evils, the nature of which their general education and observation did not allow them to understand; and secondly, that the suddenness of the war took them by surprise, and did not even permit them to bring into play such knowledge and such resources as they already possessed. We have heard these apologies made, in fact, over and over again. But of what avail are they or their like? To use the last-named excuse indicates only, if it be true, that England is a much weaker power than she would willingly admit herself to be; for a great nation, like a great man, ought never to be taken by surprise; and if the British lion cannot place *nunquam dormio* on his crest, he may soon expect the fate of his brother in the fable, and receive with feeble indignation the last blow from those whom he now most contemns. To use the first excuse, on the other hand, is but to admit the fact, that the most important and simple rules for the preservation of the health of the soldier have been altogether forgotten or neglected.

It makes this neglect, or this ignorance, still more glaring, moreover, to feel that the principles which ought to have been practically carried out in this war are all well known, having been learned in past wars after a long and bitter experience, and even reduced to writing by several competent observers. Let us dwell on this point as one of some moment, and briefly indicate how much might have been learned from the past, had the past been referred to at the time when our ill-fated expedition was first sent out.

The theoretical principles which should form the basis of every code of laws in relation to the preservation of health are now well understood, and have in previous times been accidentally put in practice without failure in results. They are simple; they are common sense itself; they are almost instinctive; and, in a pecuniary point of view, they from first to last imply a strict economy. These facts are true as regards general sanitary measures; and they are especially true, as the experience of previous wars has proved, in relation to military institutions; nor is there one cause from which the British army has suffered in health during the Crimean campaign, that has not been pointed out as the cause of similar previous disasters by one or more of the writers whose names and the titles of whose works appear at the commencement of this article.

In the first place, theory and practice have both indicated that change of climate is not very hurtful to life, if certain recognised precautions are observed, which tend to render the body capable of adapting itself to the change of circumstances under which it may be placed. This statement may seem a mere truism to most minds, since there are few who do not know and feel the necessity of modifying habits and customs, even in their own country, during the various changes of weather and of season; truism as it is, however, it is an important one to be remembered; and happy the man or the army who is wise enough not to disregard it. Let us, on this point, hear Sir John Pringle:—

> "Winter expeditions, though severe in appearance, are attended with little sickness, if the men have good shoes, quarters, fuel, and provisions. Of this we had one proof in the march into Germany, and another in that to the North in the year of the rebellion." (P. 119.)

Secondly. In reference to the lodging of wounded or sick soldiers, it has been shewn by past experience that the tent should always be avoided as much as possible; that even in the wildest districts hospital accommodation can almost always be secured; and that a church, a barrack, a line of houses, or even a cattle shed, may, under prudent regulations, be made the best of all temporary hospitals. It has further been pointed out, that the building itself, if sufficiently large and situated on a healthy spot, depends for its fitness mainly on the mode in which its beds are arranged, its supplies secured, and its apartments ventilated. On this subject, let us for a moment turn again to the writings of Sir John Pringle, who at p. 106 thus remarks:—

> "As to the description of hospitals, with regard to preserving the purity of the air, the best rule is to admit so few patients into each ward, that any one unacquainted with the danger of bad air might imagine there was room to take in double or triple the number. It will also be found a good expedient, when the ceilings are low, to remove some part of them, and to open the garret story to the tiles. It is surprising in how few days the air will be corrupted in close and crowded wards; and what makes it hard to remedy the evil, is the difficulty of convincing either the nurses, or the sick themselves, of the necessity of opening the doors or windows at any time for air. I have generally found those rooms the most healthful where, by broken windows and other wants of repair, the air could not be excluded."

Thirdly. In relation to the necessaries of life, both science and instinct have long since pointed out their simple but requisite characters. Thus, physiology has shewn with mathematical accuracy the amount of common air which every warm-blooded animal demands for the support of its existence; it has indicated the proper temperature at which the body must be kept; it has demonstrated the means by which such temperature can be best supported; and, in relation to diet, it has disclosed the important fact, that if certain mixtures or varieties of sustenance are not regularly supplied, disease and death are the inevitable results.

Fourthly. The peculiar mental trait of the English soldier, when invalided, was long ago thoroughly understood; and the evil effects of such peculiarity have often shewn themselves on previous occasions. Upon this point let us quote what Hennen said more than thirty years ago.

"There is, perhaps, no body of men more thoughtless, when left to themselves, than soldiers; they have been so long accustomed to have all their wants supplied or anticipated, and have, in fact, been so completely transformed into machines, actuated and directed by their superiors, that, if uncontrolled, they are either helpless or degenerate. It is then that one of their characteristics, while under the eye of their officers, is completely laid aside; in their absence, and in the indulgence which they suppose a residence in an hospital implies, they forget, or wilfully neglect, the most obvious means of cleanliness and regularity, and sink into filth, sloth, and debauchery. These men, the greatest part of whose lives has been passed in the open air with their corps, no sooner get within the precincts of an hospital, and beyond the immediate cognisance of their officers, than they shut up every aperture of their wards, whether accidental or constructed for the purpose of ventilation; and so long as the means of closing a window, door, fire-place, or ventilator, is left them, so assuredly do they close them up." (P. 56.)

Fifthly. The experience of the past wars has pointed out the great importance, in hospital management, of placing the beds of the patients at a slight distance above the level of the floor. This point, small as it is, seems to have been held as of the highest importance. Hennen thus writes in regard to it:—

"Every effort on the part of the medical officers should be used to procure boards and tressels, or other temporary means of removing the beds from the surface of the floor; for, independently of their comfort and cleanliness, and the prevention of damp, it is a fact now well known in military hospitals, that the lower portion of the atmosphere of the occupied ward is invariably the least proper for respiration, and that in which sores heal most slowly." (P. 59.)

Lastly. In reference to the conveyance of the wounded, and on the evil effects of over-fatigue, many eminent civil and military authors have written at great length. The effects of fatigue have indeed been fully discussed by a variety of sensible men; and it has been shewn over and over again, that excessive bodily exertion retards rather than forwards the work which it is intended to accomplish. Even the American slave-driver has found out this fact, and employs it to his advantage.

We have thus traced out and illustrated the fact, that the means most conducive to the health of an army on active service have all been faithfully described by competent observers. We can easily imagine, however, that in a campaign like the one now progressing, one or more of these regulations might by accident or unfortunate circumstances be omitted. But the peculiarity of the case is, that as the evidence delivered before Mr. Roebuck's committee shews, not one only, but every one, of these simple and common-sense principles have been overlooked. Thus, the influence of climate was ignored, and the troops were left unsheltered, and we might almost say altogether unclothed. The sick were lodged in tents, when houses were close at hand, ready and fitted to become excellent temporary hospitals. The supply of the necessaries of life was of a kind quite unfitted for the maintenance of existence. The peculiar mental character of the diseased soldier, to which Hennen so forcibly refers, was forgotten. The removal of the wounded was performed in the most clumsy, painful, and dangerous manner. The importance of placing sick men at a distance, however small, from the floors of their apartments, was unattended to. And the fatigue to which the soldiers were subjected was carried to an extent incompatible with life, and disgraceful to humanity. Looking at all this, the wonder is, not that thousands died, but that any single one, subjected to such unfavourable conditions, should have survived at all. The whole picture is made up, in fact, of a grand series of physiological phenomena, arising mainly from starvation, which, if observed and recorded faithfully by any competent man, would form a most valuable contribution to the science and practice of medicine.

Of the rottenness of the system which has led to these disastrous results, it is not our intention to speak. We believe, indeed, that individual ignorance, perverseness, moral cowardice, and want of common sense, have done far more than routine to bring about these disastrous results; and that upon the system has been laid much of the blame which ought to be heaped on the shoulders of those whose duties lie in the administration of the system. Was it from the fault of the system, that men were driven into the trenches to work for three days at a time without rest, when an equal division of work and repose would have led more quickly to the accomplishment of the objects desired? Was it mere system that placed six different men, in as many months, at the head of the hospital at Scutari? Was there any thing in the system that should lead a medical officer to refuse the use of food and necessaries to starving and dying men? Was there anything so ridiculous in the system that should prevent a capital laundry from being emptied of the chopped straw with which it was filled? Did the system make officials send home reports of the state of things, which are now proved and admitted not to be true? And, lastly, what could there be in the system that should prevent sick men from being taken into empty houses, ready at any moment to be turned into temporary hospitals? Surely, in these particulars—and they are of importance second to none other—the system is much more blameless than are its chief administrators. Had there been half a dozen such men as Mr. Macdonald (whom we hold to be the first great man that the war has brought out), connected with the military department, we believe that we should have heard very much less either of the evils of the army system, or of the appalling misery of England's warrior sons. The system is unquestionably rotten, and would disgrace even a Cretin race; and it must be reformed; but let it at the same time be remembered, that the best of all systems will fail in the hands of the uninformed, the incompetent, the irresolute, or the dishonest.

The present condition of the British army and its sanitary regulations having been thus described, two questions only remain on this occasion for discussion. How are the evils referred to, to be removed? and how is the recurrence of such evils to be prevented in the future?

The plans already commenced by the Government of establishing hospitals of ease near the seat of war, of sending out large numbers of medical men and sanitary commissioners, and of supplying provisions and necessaries with more expedition and less ceremony, are all good in their way, and will relieve temporarily the pressure of the present time. But if the war lasts long, these auxiliaries will prove cumbrous, expensive, inoperative. The army must be made complete and efficient in every department, or it will never perform the services for which it is intended.

The first step, therefore, that ought to be pursued in this dilemma would be to find, if possible, and send out, men competent as regards knowledge, and active in administration. The next step—and this is the most important in the long run—would be to introduce a remodelled system of education. As regards the medical department, the French organisation should be copied; and no medical officer should take part in the service until he has proved that his knowledge of the medical art extends to the prevention of disease as well as to its cure. As the officers appointed to command are taught the system of fortification and assault, so should the medical officer, in his way, understand the construction and management of what are really the most important fortifications of the British army—hospitals for the sick and wounded. The general principles of physiology and hygiene ought also to be taught, more or less, to every officer and every man in the service; and for the generalship of a great army, the selection should rest, not on this man's mere bravery, or on that man's tact in wielding masses of men, but on the display of a general knowledge of all that concerns the service—of the commissariat, the hospital, the discipline, and the management of troops in the field. It would be but right, also, that a government record of the health and sanitary state of the army should be regularly published; and that any great improvement, suggested by the soldier in regard to the comfort and well-being of his class, should receive its reward, as for a feat of arms in the battle-field, or for any other meritorious service. Finally, a sanitary code for the whole nation, and for the army especially, should be formed; a departure from which should be regarded as an offence against the laws of the land, and a breach of military discipline.

---

### CHRONOLOGICAL SURVEY OF THE EPIDEMICS OF EUROPE AND WESTERN ASIA, PREVIOUS TO THE ERA OF HIPPOCRATES.*

To write, or more properly speaking, to compile the histories of the numerous and various epidemic maladies which have prevailed in the world, from the earliest period of which we possess any veritable records, is no trifling task. It is a labour requiring not only deep historical research, but also a certain amount of practical experience gained in at least some of the many climes, in which epidemics have from time immemorial been rife, and to which, in fact, they may be said to be indigenous. A sojourn of a quarter of a century in countries where some of the most deadly epidemics have prevailed, has afforded us some considerable experience as to the nature of these disorders; and we hope now, by blending such personal experience with the narration of the historical facts at our own command, to be able so to write, as to lessen considerably the literary researches of succeeding writers on epidemiological subjects.

Epidemic diseases, from the vast number of persons whom they attack at one and the same time, from their intensity, and from their destructiveness, possess special claims to the earnest attention, not only of the physician or pathologist, but of all mankind. They are exceedingly interesting both in a physical and in a moral point of view; their histories, and the investigation of their causes, leading to an insight into the organisation of the world. They prove how thoroughly the sum of organic life is subject to the great

---

* BIBLIOGRAPHY.

The Pentateuch and the Historical Books of the Bible.
Capmany. Compendio Historico y Cronologico de las Pestes y Epidemias.
Casiri. Biblioteca Arabigo-Hispana Escurialense.
Mariana. Histor. Epidem.
Plutarch. Lives of Romulus and Numa.
Titi Livii Patavini Historiarum ab Urbe Conditâ, Liber Primus.
Zosimus. History of the Roman Emperors from the Time of Augustus.
Dionysius Halicarnassus. Oxford Edition, 2 vols. fol. 1764; also Edition by Reiske, 6 vols., Lips. 1774.
Murator. Epidemiologia.
Tacitus. Annals.
Homer. Iliad, Book I.
Kennet's Antiquities.
Hook. Epidemic Pestilences chronologically detailed.
Josephus. Antiquities of the Jews.

powers of nature, and how extensively all organised bodies are liable, from causes as dissimilar as they are numerous, to change and decay.

These diseases assail and carry off mankind at all times and in all places. Murrain destroys the lower animals; blight spares not the vegetable kingdom. In fact, all nature is subject, in various degrees, to devastating influences, based on immutable laws, and arising from original and supreme provisions.

Epidemic diseases, being essentially acute, and running rapidly through their stages, require not only that we should be prepared promptly to relieve those who are attacked, but also that we should be enabled by prophylactic measures to prevent the healthy from being affected. The knowledge, necessary for the accomplishment of this object, can only be acquired by an unprejudiced investigation of the atmospheric influences resulting from meteorological changes; of the endless variety in the circumstances of social life; and, in short, of all that is known of the laws by which epidemic visitations are governed, the causes whence they arise, and the conditions under which they are propagated.

The period of time, included in the dates given at the head of this article embraces what is called by some writers the mythological period of the world's history; consequently many of the facts relating to epidemics in this section of time are expressed in very obscure language, and have to be received and commented on with extreme caution. In recording them, authors were led to view matters with very different feelings to those of the present day. A strong theological bias tinges every description; and every great epidemic phenomenon is ascribed to the direct agency of a supreme power, acting not through secondary causes, but directly. The historians of most of these epidemics, indeed, belong to the theological class; having been pretty generally priests and poets, whose minds were naturally drawn from mere mundane influences to topics of a higher and more poetical character. Still, it must be admitted,—and we say this with all reverence,—that in the history of every form of pestilence occurring in this early part of the history of the world, some kind of accidental remark in the narration serves to point out to the reader of the present day, that a material cause was always at work in producing the phenomena described.

We must here offer a remark on the term "pestilence" or "plague." These terms, as used by the ancients, are meant in a general sense, to express every kind of spreading disease. Thus the Hebrew word דבר *(deber)*, the Greek λοιμος *(loimos)*, and the Latin *pestis*, are all general terms referring to any variety of epidemic.

The histories with which we are about to deal are derived from the records of not more than four nations,—the Jewish, the Grecian, the Roman, and the early Spanish. They extend over a period of nearly a thousand years,—that is to say, from the time of the Exodus, 1491 B.C., to the overthrow of the regal power in

Rome, and the establishment of the consulate in the persons of Brutus and Collatinus, 508 B.C. The whole period was one of war and semi-barbarism; and was the theatre of many of those great events which form the basis of our modern European development. Thus it takes in the various phases of progress in the Hebrew nation, the expedition of the Argonauts, the establishment and partial decline of the Grecian states, the Trojan war, the foundation of Rome, and its period of regal government.

Perhaps the first entries of any epidemic are those recorded in the Mosaic books, as occurring in the reign of Pharaoh, king of Egypt, 1491 B.C. Two distinct epidemics are recorded as having affected the Egyptians; viz.—ulcerous eruptions or boils, and a pestilence which, in one sudden and universal destruction, swept away thousands of the inhabitants of that clime. The first was preceded in succession by a remarkable change in the water of the Nile, probably by the generation of a peculiar insect, by the appearance of extraordinary numbers of frogs, vermin and flies, and by a murrain among cattle. The second epidemic occurred about the month of March, and was preceded by remarkable meteorological phenomena. The weather had been variable; excessive heats and hot burning winds had exhausted the inhabitants by day, while cold damp dews chilled them by night. This alternation from suffocating heat to damp cold, preceded by universal darkness (occuring on the 10th), attended with a fearful commotion of the elements, evidenced by hail, thunder, and lightning, with want of water, occasioned by long droughts, and the generation of vast quantities of insects, ended in the ushering in, on the 14th, of a deadly pestilence.

About the year 1490 B.C., another very interesting epidemic occurred among the Israelites, occasioned by their eating great quantities of the flesh of quails, to which diet they must have been unaccustomed, since for some time past they had been destitute of animal food. The nature of the epidemic is not described; but it is almost inevitable, that, under the circumstances, the voracious eating of animal flesh in such hot climates as Egypt and Arabia would lead to some kind of intestinal disorder.

By another recorded pestilence, 14,700 Israelites were carried off, on the occasion of the mutiny of Korah, Dathan, and Abiram, in the encampment at Kadesh, B.C. 1471.

About nineteen years afterwards, B.C. 1452, by a severe epidemic among the riotous and drunken worshippers of Baal-peor, 24,000 subjects, male and female, perished.

In the year B.C. 1310, the island of Ægina was nearly depopulated by severe epidemic disease.

In the latter period of the Hebrew commonwealth, which terminated B.C. 1095, we have an account of an epidemic occurring among the Philistines, by which 50,000 perished at Bethshemesh. The pestilence here referred to is styled *emerods*, and was, according to Josephus, of a dysenteric type; and it is curious to observe, that it occurred at a time of the year when dysentery is a common epidemic. It appears that the ark of the covenant was in the hands of the Philistines seven months, and returned during wheat harvest. The crops, having been sown in October and November, were reaped about May; soon after which the plague seems to have ceased; and it may be presumed that the pestilence was most violent in the latter part of April and the early part of May. This account corresponds with what has been observed in modern times.*

Omitting the mythical account of the epidemic among animals and men referred to by Homer, in the first book of the Iliad, the next notice of epidemic disease is of one occurring in Spain. The period of this epidemic is variously given; by some it has been fixed at 1100 B.C., while others† mention it as having occurred during the first plague in Egypt. Whatever the date may be, however, it seems that, for twenty-five years previous to the plague, a great drought prevailed in Spain. Springs were dried up, rivers became fordable; there was neither food for beasts nor man; and so great was the barrenness of the land, that scarcely any green thing was to be found, except some olive trees on the banks of the Ebro and Guadalquiver. "Such," says the historian, "was the melancholy state of our ancient Spain, full of mortalities, plagues, and miseries of every description; which, together with emigration, depopulated the country."‡

It was after this time, in the year 1017 B.C., that a pestilence, destroying 70,000 people, occurred among the Israelites, during the reign of David. This pestilence is attributed to the numbering of the people, but its nature and physical cause are not described.

We know of no other historical note on epidemics until the foundation of Rome. Plutarch, in his Life of Romulus, refers to the first plague at Rome. From his description, it appears that Rome had for some time previously suffered from atmospheric pollution and from varieties of season. The country around was marshy, near the Tiber, and was overcrowded. The epidemic occurred about the sixteenth year after the foundation of Rome (B.C. 738), soon after the murder of Tatius. It was ushered in suddenly; men, animals, and even vegetable life, suffered; and all nature lay as one abandoned waste. Blood was said to have been rained, as in the plagues of Egypt. Another writer (Zonaras) also refers to the fact, that Rome was laid waste by disease, that the earth became barren, and that even cattle suffered—"*sterilitas agrorum et pecudum.*"

In the year B.C. 710, during the reigns of Numa Pompilius in Rome, of Hezekiah in Judah, and of Sennacherib in Assyria, an epidemic, also described by Plutarch, afflicted the inhabitants of

---

* Russell's Plague of Aleppo.

† Capmany, Compendio Historico y Cronologico de las Pestes y Epidemias, tomo iv. de las Memorias Historicas, num. 7, p. 66.

‡ Casiri, Biblioteca Arabigo-Hispana Escurialense, tom. ii., pp. 71 and 72, 89, 334. Mariana, lib. ii., cap. 2, 3, 4, 6, 10, 13; lib. iii., cap. 2, 3, 6; lib. iv., cap. 28 and 44; lib. v., cap. 8.

Italy.* A pestilence also, at this time, destroyed 185,000 of the Assyrian soldiery, who were besieging Jerusalem under Sennacherib.

Rome again suffered, as described by Livy (book i. ch. 32), from a severe epidemic, which occurred during the reign of Tullus Hostilius, B.C. 640 or 645. Zosimus, an officer during the reign of Theodosius the Younger, gives an account of a pestilence in Rome, which afflicted its inhabitants during the reign of Tarquin, B.C. 594;† but Hook records this violent epidemic as having occurred B.C. 514.‡

Dionysius Halicarnassus, also Murator and Functius,§ mention the occurrence of famine, succeeded by pestilence, in Rome, B.C. 545, which nearly depopulated Velitiæ or Veliterna, an ancient town of Latium on the Appian Road, about twenty miles to the east of Rome. A murrain also prevailed among cattle in the Roman States, which caused great havoc among them, as it did among the inhabitants of Latium, to the extent that they were compelled to re-people their cities by application to Rome, which state also suffered the subsequent year. It also prevailed in Campania. This pestilence spared neither age nor sex, or even constitution; and it would appear to have been so deadly, that it yielded to no remedies. It appeared suddenly; destroyed its victims rapidly; and, on the approach and continuance of cold weather, disappeared, it might be said, as suddenly as it had appeared.

Reviewing, then, what we have written, it seems that, from the time of the Exodus of the Israelites to the period immediately preceding the advent of Hippocrates, at least fourteen great epidemic visitations may be traced out in history. The inference, of course, is, that in so long an epoch many other epidemics occurred, which have escaped notice. We believe that the records of such visitations as have been referred to are generally authentic. What the special symptoms were, we may not at first sight be able to recognise in accordance with our modern nomenclature; nevertheless, by careful perusal and investigation, we are enabled to identify some of them sufficiently to shew that the ancients were accurate observers of nature and of nature's laws, and to prove the superiority of their arrangements as regards nosological distinction and classification, which were simple, and thus prevented the minds of observers from being divested from the true characters of disease. Our inability to trace diseases under the names and characters described by our predecessors in the study of nature may be said to depend on the indistinctness of the descriptions given; and this has arisen both from false or imperfect translations, and also from

---

* Plutarch's Life of Numa. Kennet's Antiquities, book ii., part 2.

† Zosimus, lib. 2.

‡ Vol. i., p. 109.

§ Murat., tom. i., chap. 5. Also Hook, vol. i., page 196; Funct. Chronol.; Dion. Halicar., lib. 7.

the practice of the ancients in referring different malignant maladies to the same pestilential constitution; for it must be remembered that they considered all febrile diseases to bear a close affinity to each other, and hence classed all pestilential epidemic distempers under a general term—*pestilence, plague,* or *fever;* under the head of *consumption,* they classed all chronic diseases; and boils, scabs, pustules, blotches, carbuncles, &c., were included under the name of *skin-diseases.* Further, two facts of great interest may be extracted from what is recorded,—viz., that epidemic disorders, in men, animals, and plants, are common to all times and all nations; and that many of the causes which gave rise to them in the remotest periods of the world's history are identical with those which excite them now—impure air, overcrowding, uncleanliness, famine, and the manifestation of peculiar meteorological conditions.

---

### (B.—ANALYTICAL.)

### ON THE APPLICATIONS OF CHARCOAL TO SANITARY PURPOSES.

A Lecture. By JOHN STENHOUSE, LL.D., F.R.S., Lecturer on Chemistry at St. Bartholomew's Hospital, London. London: Samuel Highley.

THIS little work, from the pen of the distinguished Professor of Chemistry at St. Bartholomew's Hospital, is written to explain the value of charcoal as a deodoriser and disinfectant. After describing the varieties and manufactures of charcoal, the author proves, from careful experimental researches, the effects of various kinds of charcoal in absorbing deleterious gases. Animal charcoal absorbs least of these gases,—peat charcoal more, wood charcoal most. In speaking of this point, Dr. Stenhouse tells the following curious anecdote:—

"Great efforts have been recently made, chiefly in Ireland, however, to persuade the public into a belief of the superior efficacy of *peat* charcoal for sanitary purposes. A single glance at the table shews that this is not warranted by the fact, and that peat charcoal is slightly inferior, as an absorbent, to ordinary wood charcoal. Notwithstanding this, however, I lately saw it ostentatiously announced in the newspapers, that thirty tons of peat charcoal had been sent to Scutari, for the use of hospitals in Turkey, by the Irish Amelioration Society, who did not appear to be at all aware that wood charcoal is the ordinary fuel employed in Turkey and most other Eastern countries, where it can always be had of the best quality and in any quantity that may be desired.

"This proceeding with regard to the peat charcoal reminds one of the old proverb of "carrying coals to Newcastle," though, unfortunately, it is but too much of a piece with most of our doings in regard to our hitherto ill-starred expedition to the Crimea."

The uses of charcoal for the purification of water has long been known. Dr. Stenhouse carries this principle much further, He shews that charcoal as a general deodoriser and disinfectant of the first class,—that it is, in short, the best of all agents for purifying the foul air. The origin of his inquiries are thus described:—

"My attention was particularly drawn to the importance of charcoal as a deodorising and disinfecting agent, about eighteen months ago, by my friend,

John Turnbull, Esq., chemical manufacturer, of Glasgow. Mr. Turnbull, about six months previously, had placed the bodies of two dogs in a wooden box, on a layer of charcoal powder of a few inches in depth, and covered them over with a quantity of the same material. Though the box was quite open, and kept in his laboratory, no effluvia was ever perceptible; and, on examining the bodies of the animals at the end of six months, they were found to be in a very advanced state of decay. Mr. Turnbull sent me a portion of the charcoal powder which had been most closely in contact with the bodies of the dogs. I submitted it for examination to one of my pupils, Mr. Turner, who found it contained comparatively little ammonia, not a trace of sulphuretted hydrogen, but very appreciable quantities of nitric and sulphuric acids, with acid phosphate of lime. Nearly eighteen months ago, I buried the bodies of a full-grown cat and two rats in about two inches of charcoal powder, and kept them ever since in my laboratory. During the whole of this time not the slightest odour has been perceptible, nor have any injurious effects been experienced by the eight or nine persons by whom the laboratory is daily frequented. On recently examining the state of the animals, I found that almost all the nitrogenous portions had disappeared, and that what remained consisted chiefly of bones and a portion of fat, and even this latter substance was in a state of rapid decay."

The author next points out, with great accuracy, that the idea expressed by almost all our chemical authorities as to the antiseptic properties of charcoal, is simply contrary to facts. Charcoal, from the considerable amount of oxygen contained in its pores, not only absorbs, but rapidly oxidises the effluvia and miasmata emitted by decaying substances, and resolves them into their simplest combinations, a process which renders them inert as animal poisons, by causing them to undergo a process of low combustion, which as effectually destroys them as though they were passed through a furnace.

Reflection on the effects of charcoal as a deodoriser led Dr. Stenhouse to think of applying this substance to a practical purpose, in presenting the injurious effects which result from the minute quantity of putrid infectious matters floating in the air of unhealthy places. This led further to the invention of what he calls his Charcoal Air Filter.

"It consists of a thin layer of charcoal powder, interposed between two sheets of wire gauze, and can be readily applied to buildings, to ships, to gully-holes of sewers, to respirators, and to various other purposes. One of these charcoal air-filters was fitted up in the justice-room of the Mansion-House, about three months ago, where it has ever since been in successful operation."

The application of this filter admits of very wide application. It might be inserted in the framework of ships, in the wards of hospitals, in narrow streets, in mews, in the gulley holes of sewers, and other similar places. It would be useful in pestiferous districts, and in the sick room. The following is the kind of filter now in use:—

"The air filters, or charcoal ventilators, at the Mansion-house and Guildhall are each of them several feet in diameter. The layer of charcoal is about an inch and a half in thickness, and consists of fragments from the size of a pea to that of a largish bean."

From the description of the air filter, the author passes to the consideration of charcoal respirators. Of these there are three forms. The first is for the mouth, the second for the mouth and nose, and the third is also for the mouth and nose, but is of a large size. In persons wearing these respirators no air enters into the lungs without first passing through the charcoal, and thus all effluvia are destroyed. The charcoal respirator is stated to have three advantages :—

"1stly. Where the breath is at all fetid, which is usually the case in diseases of the chest, under many forms of dyspepsia, &c., the disagreeable effluvia are absorbed by the charcoal, so that comparatively pure air alone is inspired.

"This, I think, may occasionally exert a beneficial influence on diseases of the throat and lungs.

"2ndly. The charcoal respirator for the mouth alone will certainly prove highly useful in poisonous atmospheres, where miasmata abound, if the simple precaution is only observed of inspiring the air by the mouth, and expiring it by the nostrils.

"The charcoal respirator is exceedingly easy to breathe through, as, owing to the non-conducting nature of the material of which it consists, it does not condense the moisture of the breath to an inconvenient extent."

The charcoal respirator would, in the opinion of this author, entirely prevent painter's colic ; the evil effects arising from the explosion of guns in case-mated batteries ; and the mischief which often results to Indian travellers on passing through regions where unhealthy exhalations, from putrifying vegetable matter, abound. At page 26, he tells another anecdote whlch certainly deserves notice.—

"A few days ago, during an interview which I had with Dr. Sutherland, who has just gone out as medical inspector to Scutari and Balaklava ; that gentleman informed me, that so strongly was he convinced of the utility of charcoal respirators, that he had memorialised Government to allow him to take out 500. Dr. Sutherland's request was met with the usual stereotyped official reply, "that respirators did not belong to his department." Dr. Sutherland was, therefore, obliged to content himself with taking out a single dozen for the use of himself and his brother inspectors."

Dr. Stenhouse concludes his interesting pamphlet with an enthusiastic prediction, that the time is nearly come when the propogation of disease by infection will be the exception, not the rule. *Speramus.*

---

### GYMNASTICS AN ESSENTIAL BRANCH OF EDUCATION, BOTH PUBLIC AND PRIVATE.

By Captain Chiosso, Professor of Gymnastics at University College School, London. pp. 72. London: 1854.

One of the elements in the pharmacopœia of health is *bodily exercise ;* and it is Captain Chiosso's object to shew the importance of this element, and the manner in which it can be best applied to improving the condition of the community. While the ancient Greeks and Romans pursued gymnastic exercises as a branch of education, and while there is abundant evidence of the superior healthy condition which attends occupations involving a due amount of muscular exercise, it is evident, on the other hand, that the practice of gymnastic exercises has fallen too much into disuse among us, and that *mental* has too far taken the lead of *physical* education.

Some of the implements and exercises of the modern gymnasium having been described, the author lays down rules and precautions to be observed, the neglect of which has, in many instances, brought unmerited odium on gymnastics. These rules and precautions refer to the health, habits, temperance, time of day at which the exercise should be performed, the degree and amount of exercise, &c. From the adoption of a system of gymnastics thus properly regulated, Captain Chiosso anticipates great and happy changes in the condition of the people.

"It is *gymnastics* (or *nature*, or *muscular life* of any kind) which will be the only preventive, lest the other pole of life" (the nervous as the author calls it) "should take the ascendancy. * * * Happy the child whose parents have become impressed with this truth! Happy the young person who has arrived early at this conviction! Still happy they who, at any period of life become aware of this great truth, and thus are enabled to impart greater force and duration to even the last sparks of their life! Happy, indeed, in fine, will be the country where this great radius of the life of the *ancients* will be added to the advantages of *modern civilisation*, without which (addition) such advantages are, after all, useless. It will be a great epoch of modern history, when any sovereign or government shall have ordered the erection of gymnasiums (lesser or larger) in every open space of our town and cities, in our parks and gardens, and on our commons, to which, early and late, a serene, innocent, and happy youth, may resort; when, in every country, a central gymnastic establishment for the training of masters shall have been established; when, in fine, a satisfactory testimonial from a gymnasium will be considered as *essential* as that of any other educational department." (pp. 66-7.)

Captain Chiosso's suggestions are worthy of the attention of all who are interested in the improvement of the physical and of the moral condition of the community.

---

### REPORT OF THE COUNCIL OF THE BRITISH METEOROLOGICAL SOCIETY.

Read at the Fourth Annual General Meeting, May 23rd, 1854.
Pamphlet, pp. 34.

THIS Report gives a summary of the proceedings of the Society during the preceding year; and it also furnishes us with the gratifying information, that meteorology is becoming recognised as an important branch of study in other countries. In Brussels, the subject has received the attention of the Royal Academy of Sciences; and

"Under M. Quetelet, at various places on the globe, a regularly organised system has been established for simultaneous observations relating to the leafing, flowering, and fructification of trees, shrubs, &c.; the departure and return of migratory birds; the torpidity, reappearance, and disappearance of animalculæ, insects, reptiles, &c.; for the formation, in fact, of a calendar of nature, which, based upon simultaneous observations over a large portion of the earth's surface, will open the influence of the seasons in particular, and of geographical position in general, in accelerating or retarding the return of these periodical phenomena; with the view also of arriving at correct conclusions regarding what may be termed normal conditions of the air, in opposition to those which are abnormal, and are found to act injuriously upon the animal and vegetable kingdoms."

The Society had also received information that a number of stations for meteorological observation had been established in Spain. It is to be hoped that these stations are under the superintendence of observers who are competent, not merely to register abstract meteorological facts, but also to observe and record their connection with the phenomena of health and disease.

The papers read before the Society during the year, and of which abstracts are given, viz.—

"The Meteorology of the Quarter ending December 31st, 1853, and the Beginning of the Year, 1854." By James Glaisher, Esq., F.R.S.

"Yearly Meteorological Report for 1852." By Charles Smallwood, Esq., M.D., St. Martin, Isle Jesus, Canada East.

"Medico-Meteorology and Atmospheric Ozone." By Dr. Moffat.

"A Certain Law in the Direction of the Wind, together with a map exhibiting the results of 2000 Observations." By Charles Bulard, Esq., B.A.

"The Fall of Rain in the Years 1852 and 1853." By James Glaisher, Esq., F.R.S.

"Meteorological Observations made at St. Martin's, near Montreal, Canada East." By Charles Smallwood, M.D.

The most important part of this report, in relation to disease, is furnished by Dr. Moffatt, who infers, from his laborious inquiries, "that the maximum of disease occurs with directions from the south or ozone points, and the maximum of deaths with directions from the north, or no ozone points; and that some diseases are peculiar to certain points of the compass. Cases of diarrhœa, influenza, premature labour, toothache, neuralgia, epilepsy, and sudden death, are said to be almost peculiar to winds varying from the north to the north-east and east.

